# Burden and Epidemiology of Human Intestinal *Giardia duodenalis* Infection in Colombia: A Systematic Review

**DOI:** 10.3390/tropicalmed7100325

**Published:** 2022-10-21

**Authors:** Carmine Fusaro, Yosef A. Chávez-Romero, Sonia Liliana Gómez Prada, Nancy Serrano-Silva, Jaime E. Bernal, Francisco Erik González-Jiménez, Yohanna Sarria-Guzmán

**Affiliations:** 1 Facultad de Ingenierías, Universidad de San Buenaventura, Cartagena de Indias 130010, Colombia; 2 Facultad de Estudios Superiores Zaragoza, Universidad Nacional Autónoma de México, Santa Cruz 90640, Mexico; 3 Facultad de Ciencias, Universidad Nacional Autónoma de México, Mexico City 04510, Mexico; 4 Facultad de Medicina, Universidad del Sinú, Cartagena de Indias 130011, Colombia; 5 Facultad de Ciencias Químicas, Universidad Veracruzana, Orizaba 94340, Mexico; 6 Facultad de Ingeniería y Ciencias Básicas, Fundación Universitaria del Área Andina, Valledupar 200005, Colombia

**Keywords:** *Giardia*, Giardiasis, Colombia, systematic review

## Abstract

The genus *Giardia* is a unicellular protozoan able to parasitize both humans and animals. Cysts of *Giardia* can be found in soil samples, aquatic environments, food, and any surface that gets in contact with the feces of parasitized animals. The aim of this systematic review was to analyze the burden and epidemiology of *Giardia* infection in Colombia summarizing recent scientific reports and existing knowledge and to identify knowledge gaps that may be addressed in future investigations. This work follows the guidelines established by “Preferred Reporting Items for Systematic Reviews and Meta-Analyzes” (PRISMA). Published scientific literature from 1 January 2010 to 18 September 2022 was searched in six electronic scientific databases using the search terms: “*Giardia*” OR “Giardiasis” AND “Colombia”. Twenty-three scientific articles were performed in 22 departments of Colombia at rural, urban, and a combination of rural and urban contexts. The prevalence of *Giardia* in the Colombian population was between 0.9 and 48.1% when the samples were analyzed with classical microscopy; the range of *Giardia* prevalence was even bigger (4.2–100%) when qPCR and nested PCR were used. The dominant *Giardia* assemblages found in Colombia were A and B, and most frequent subassemblages were AII, BIII, and BIV.

## 1. Introduction

*Giardia duodenalis* (syn. *Giardia intestinalis*, *Giardia lamblia*) is a cosmopolitan flagellated, microscopic protozoan parasite [1,2,3] able to infect a great diversity of domestic and wild animals [4,5,6]. *Giardia* spp. cysts are capable of maintaining their viability for a long time outside their host [7]. Eight *Giardia* spp. variants or genotypes, identified with the letters A–H, have been recognized to date [8,9]. Two of them, specifically the A and B genotypes, are frequently found in humans and in many animal species including cats, dogs, sheep, chickens, horses, pigs, and cows [10,11]. More recently, novel genotypes E and F have also been found in humans in Australia and Slovenia [12,13]. Parasite transmission among people generally occurs by the fecal–oral route i.e., consuming water or food [1,14].

The Giardiasis was, for a long time, an underrated and under-attended disease despite the large number of cases worldwide, probably because most people are asymptomatic or only present diarrhea as the most notable symptom [15]. Citizens living in developing countries, such as those located in the Caribbean and Latin America, with deficient water sanitary supply services and inadequate wastewater treatments, are particularly exposed to the health risk derived from a *Giardia* spp. infection [16,17]. Colombia, as well as several other tropical countries, presents the ideal geoclimatic and epidemiological conditions for the transmission of intestinal parasites such as *Giardia* spp. [18]. People living in Colombian rural areas or in the suburbs of main cities with scarce economic resources, poor water quality, and deficient hygienic conditions are the most exposed to parasitic infection [19].

The main detonators of Giardiasis among Colombians are inadequate health conditions and food risk. Colombia has greatly improved the quality services of its health care system in the last decades; data indicate that nearly 97% of Colombians have access to basic medical care [20,21]. Nevertheless, research in this field indicates that barriers and burdens to accessing high-quality health care services persist [21]. Different socio-economic, geographical, and cultural barriers affect the efficiency and readiness of the health system [21,22,23]. All these relevant features make Colombian individuals particularly vulnerable to the transmission of intestinal parasites such as *Giardia*.

According to Rodríguez-Morales et al. [24] a total of 15,851 infection cases of *Giardia* spp. were detected between 2009 and 2013 in Colombia, approx. 3300 infections per year; the capital city of Bogotá and the departments of Antioquia, Atlántico, and Risaralda presented the higher incidence rates of *Giardia* spp. infection in their citizens. Also, the results of Bedoya-Arias et al. [25], between 2009 and 2016, showed that the incidence rates of *Giardia* spp. infection in the Colombian population varied from 13.35 to 183.69 cases per 100,000 inhabitants.

Parasitic infections and diarrheal diseases are significant threats to the Colombian heath system and, in general, to all developing countries [26], causing work and school absenteeism with adverse socio-economic impacts [27,28]. *Giardia* spp. infection has effects on quality of life by causing discomfort and pain to the patients. Strategies to prevent *Giardia* infection are based on good hygiene practices, health education [29], and, no less important, the early detection of and monitoring plans for the parasite in human populations. Unfortunately, the economic recession and the increase in poverty, due to the COVID-19 pandemic, have further deteriorated the living conditions of vulnerable Colombian populations; these aspects could produce a medium-term increase of the incidence and prevalence of Giardiasis and other gastrointestinal infections.

The diagnosis of intestinal parasites such as *Giardia* spp. in human stool samples is carried out by using concentration methods plus microscopy [30] or using molecular techniques i.e., polymerase chain reaction (PCR), nested PCR, and quantitative PCR [1,31,32]. Microscopy is time-consuming and laborious [33] but cheaper, and it remains one of the most widely used methods in Latin America.

Only a comprehensive and multidisciplinary management is effective in the control or elimination of parasitic neglected tropical diseases. Classical microscopy and molecular parasitology analysis should be integrated with projects in human and social sciences fields to achieve the sustainable control of endemic parasites and improve the life quality of the individuals [34].

The aim of this systematic review was to analyze the burden and epidemiology of *Giardia* infection in Colombia by summarizing recent scientific reports and existing knowledge.

## 2. Materials and Methods

The systematic review was conducted following the standardized method of “Preferred Reporting Items for Systematic Reviews and Meta-Analyses” (PRISMA) guidelines and the checklist of Moher et al. [35]. Table S1 presents the PRISMA checklist of this study.

### 2.1. Search Strategy

The search for specific scientific literature published from 1 January 2010 to 18 September 2022 was carried out on 19 September 2022 by an author (YSG). Six electronic scientific databases i.e., ISI Web of Science (Clarivate Analytics), EMBASE (Elsevier), Science Direct (Elsevier), Scopus (Elsevier), SciELO (São Paulo Research Foundation—FAPESP), and PubMed (National Library of Medicine of USA—NLM) were employed individually to identify relevant full-text articles using the following search terms: “*Giardia*” OR “Giardiasis” AND “Colombia”. All possible combinations were sought and examined.

### 2.2. Inclusion and Exclusion Criteria

The inclusion criteria, applied to full-texts for assessing their eligibility, were: (a) original article focusing on the identification of *Giardia* spp. in Colombia, (b) article published from 1 January 2010 to 18 September 2022, (c) article written in English or Spanish, (d) study limited to human beings, (e) cross-sectional study, (f) article published in peer-reviewed journals in the Scimago Quartiles database.

The exclusion criteria, applied to full-texts for assessing their eligibility, were: (a) article published in a non-peer-reviewed source, (b) review of the literature or meta-analyses, (c) retrospective study, (d) short communication, (e) study with a score below three points based on the Joanna Briggs Institute (JBI) tool [36].

### 2.3. Selection of Studies

The identified articles were compiled using the Mendeley Desktop Reference Management System 1.19.8 and any duplicates were removed. Subsequently, two authors (YSG and CF) independently screened titles and abstracts. Irrelevant titles were removed. A third author (YCR) made a final decision when the two researchers had differing opinions. Inclusion and exclusion criteria were applied to full-texts to assess their eligibility; two authors (YSG and CF) independently analyzed the full-texts and only those that met all criteria were finally selected. Disagreements between the two researchers were resolved through consultation with a third author (YCR).

### 2.4. Data Extraction and Analysis

Article-level data were extracted from each selected paper; subsequently, they were summarized and tabulated in an abstraction-analysis matrix developed in MS Excel^®^ (Microsoft for Windows). The summarized information was organized in columns with the following subjects: (a) Reference, (b) Quartile, (c) Location, (d) Rural/Urban, (e) Collection period, (f) Study population/Group studied, (g) Age, (h) Feces samples, (i) Sample size, (j) Number of replicas, (k) Concentration method, (l) Detection method, (m) *Giardia* spp. genes, (n) Assemblages or subassemblages, (o) Prevalence (microscopy), (p) 95% confidence interval (microscopy), (q) Prevalence (molecular detection), (r) 95% confidence interval (molecular detection) and finally, (s) Study quality based on JBI tool.

### 2.5. Quality Assessment

The quality of the included studies was assessed with standardized critical appraisal instruments from the JBI [36]. The checklist consists of nine items, each one with four options (yes, no, unclear, and not applicable). The JBI score rating system divides the studies in two groups i.e., high quality studies (scores between 7 and 9), moderate quality studies (scores between 4 and 6).

Two researchers (YSG and CF) worked independently to analyze the selected material and, disagreements were resolved through consultation with a third author (YCR). The quality assessment results are presented in Table S2.

## 3. Results

### 3.1. Literature Search

**Figure 1:**
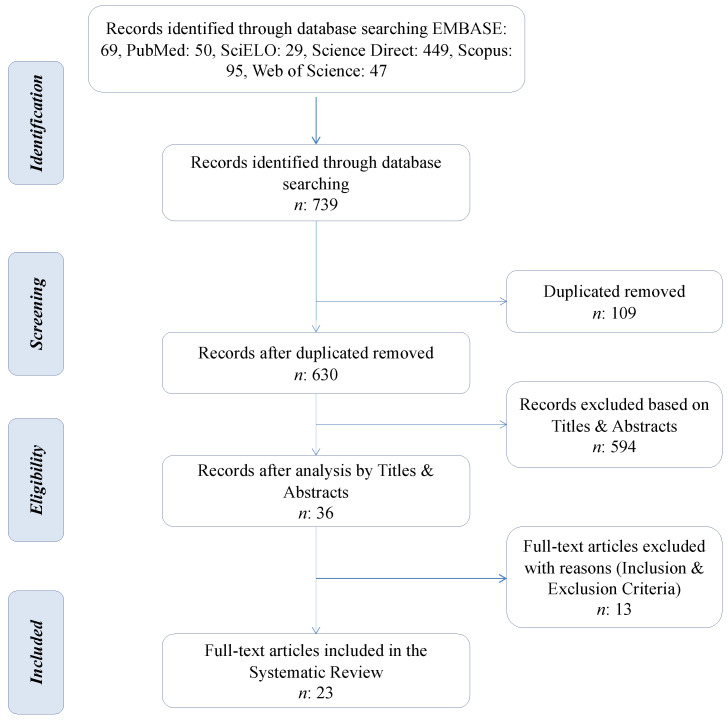
PRISMA flow diagram.

A total of 739 publications were recorded in the identification phase. Duplicates were removed and the remaining 630 articles were screened for title and abstract pertinence. Only 36 full-text articles have passed the screening of title and abstract phase. Hence, their eligibility was assessed based on inclusion and exclusion criteria. Finally, 23 articles were included in this systematic review [37,38,39,40,41,42,43,44,45,46,47,48,49,50,51,52,53,54,55,56,57,58,59]. The PRISMA Statement flow diagram, composed of four phases (identification, screening, eligibility, and inclusion) is shown in Figure 1.

### 3.2. Characteristics of Included Studies

General characteristics of the selected articles are summarized in Table 1.

All selected articles were published in journals belonging to the Scimago Journal Ranking (SJR); more specifically, six articles were published in Q1 SJR journals [46,47,52,54,55,56], four articles in Q2 SJR journals [38,44,50,57], another five articles in Q3 SJR journals [41,48,49,51,58], and eight articles were found in a Q4 SJR journal [37,39,40,42,43,45,53,59]. Based on the JBI score rating system tool, 10 of the 23 selected articles were considered as high-quality scientific papers [38,44,46,47,50,52,54,55,56,57], while the other 13 studies were of moderate quality [37,39,40,41,42,43,45,48,49,51,53,58,59].

All these papers, mainly cross-sectional studies, analyzed the prevalence of *Giardia* spp. in various social groups of Colombians. Twelve studies were performed in rural settings [37,42,44,45,46,48,51,52,56,57,58,59], eight studies were conducted on urban citizens [38,39,40,41,43,47,49,53], and the last three studies were based on a specific combination of rural and urban people [50,54,55].

**Figure 2:**
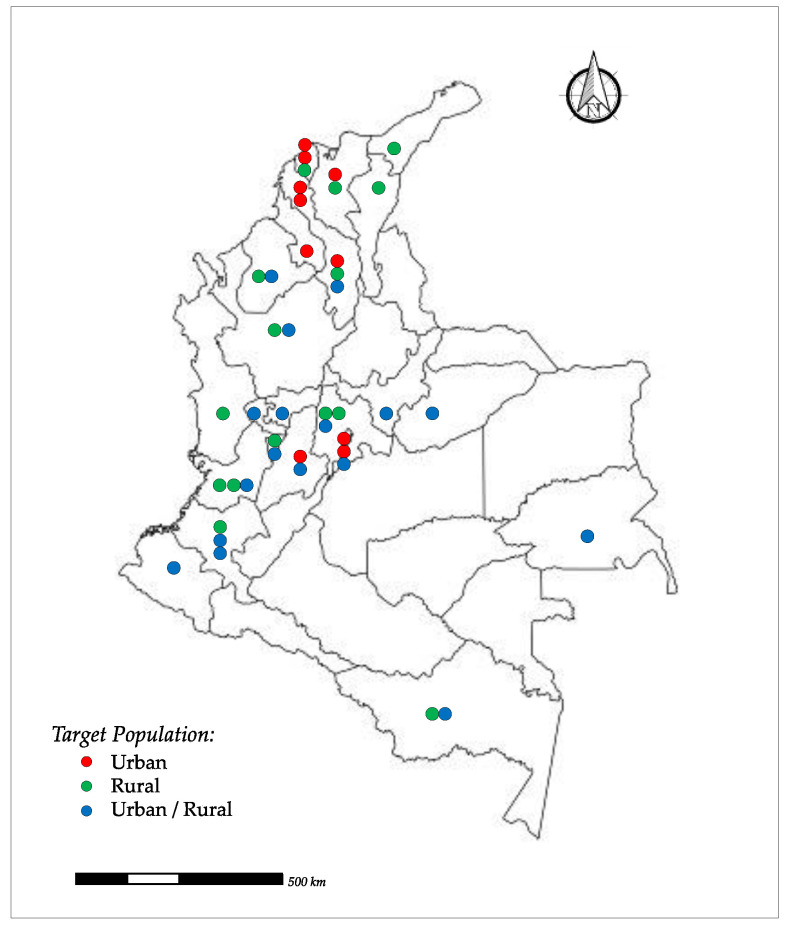
Study areas and target populations.

Scientific articles performed in 22 departments of Colombia; the bibliographic search has not yielded data in some departments i.e., North of Santander, Santander, Arauca, Vichada, Meta, Guaviare, Vaupés, Caquetá, and Putumayo (Figure 2).

Approx. three-quarters of the papers (18 out of 23) were conducted on children or teenagers [37,38,39,40,41,43,44,45,46,48,50,52,53,54,56,57,58,59], *Giardia* infection in adults was investigated by Villalba-Vizcaíno et al. [49], Carvajal-Restrepo et al. [51] and *Higuera* et al. [55]. The indigenous population was studied by Espinosa-Muñoz et al. [42] and Kann et al. [57]. Finally, the study of Espinosa Aranzales et al. [47] was conducted on pregnant women.

### 3.3. Molecular Characteristics of the Selected Studies

The main characteristics of the applied experimental methodologies can be reviewed in Table 2.

In all the studies, fecal samples were analyzed to identify *Giardia*.

Most authors did not report the number of fecal replicas used in their analysis, only Arias et al. [37], Villafañe-Ferrer and Pinilla-Pérez [45], and Muñoz Salas et al. [58] indicated that the experiments were performed in triplicate.

The most common concentration methods, designed to separate protozoan cysts from excess fecal debris, were the formol-ether technique or Ritchie concentration [37,38,39,43,44,45,47,48,55,56,58]. However, other concentration techniques such as the Kato-katz and Richie-Frick [44], Diethyl-ether method [49], Biphasic sedimentation [50], Mini Parasep SF fecal parasite concentrator [42,52], and Sheather technique [56] have been used routinely.

Generally, *Giardia* spp. was identified through a microscope exclusively or by using microscopy combined with molecular methods. Optical microscopy was performed in approx. two-fifths of the selected studies [37,38,39,42,43,45,48,51,53,56,59], the remaining twelve papers used different molecular approaches such PCR [49,55,57,58], nested or semi-nested PCR [40,44,50,52], PCR-RFLP (Restriction Fragment Length Polymorphism), and quantitative PCR [41,46,47,54].

Extraction and detection of *Giardia* spp. was carried out by means of commercial kits such as (1) Norgen Stool DNA Isolation Kit (Norgen Biotek Corporation, Thorold, Canada) [46,47,54,55], (2) QIAamp^®^ Fast DNA Stool Mini Kit (Qiagen, Hilden, Germany) [41,44,50,52,57], (3) ISOLATE II Genomic DNA Kit Cat.:137 BIO-52066 (Bioline) [49], and (4) AccuPrept Stool Genomic DNA kit (BioNeer Corp., Munpyeong-seo, Republic of Korea) [58], that were convenient and rapid methods to isolate total DNA from fresh or frozen stool samples.

Four genes of *Giardia* spp. i.e., beta-giardin (*bg*); glutamate dehydrogenase (*gdh*); triose phosphate isomerase (*tpi*), and small-subunit (*SSU*)/18S rDNA were detected among Colombians.

The assemblages of *Giardia* spp. identified were mainly A and B [40,41,44,46,49,50,52,54,55,58]. Mixed infections assemblages A + B were reported by Ramírez et al. [44], Hernández et al. [52], and Muñoz Salas et al. [58], finally assemblages D and G were reported by Villamizar et al. [54] and Higuera et al. [55].

### 3.4. Reported Prevalence of Giardiasis

The size of studied groups ranged between 23 [50] and 649 [55] individuals. The estimated prevalence of *Giardia* based on molecular identification methods was generally higher than the prevalence obtained with microscopy methods.

**Figure 3:**
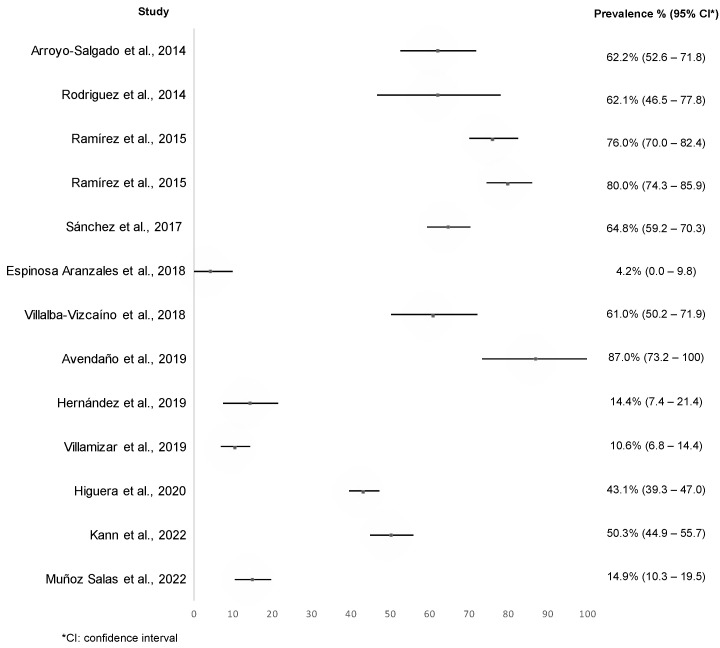
Reported prevalence of *Giardia* in the included studies by molecular method [40,41,44,46,47,49,50,52,54,55,57,58].

The prevalence of *Giardia* in the fecal samples analyzed with molecular methods ranged between 4.2% (C.I. 0–9.8%) [47] and 87.0% (C.I. 73.2–100%) [50] (Figure 3). Of the 12 investigations that used molecular methods, 8 papers showed a prevalence of *Giardia* above 43.0% [40,41,44,46,49,50,55,57]. Ramírez et al. [44], Sánchez et al. [46], and Avendaño et al. [50] investigated rural populations or mixed population (urban/rural) and reported the higher prevalence of *Giardia*; while the lower prevalence of *Giardia* was (4.2%) found by Espinosa Aranzales et al. [47] that analyzed the urban population, specifically pregnant women from Bogota city.

**Figure 4:**
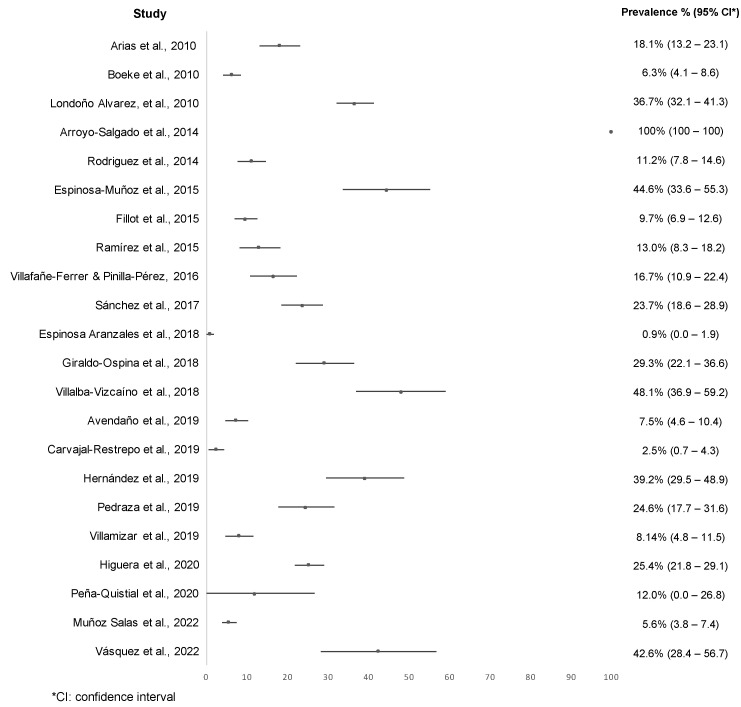
Reported prevalence of *Giardia* in the included studies by microscopy [37,38,39,40,41,42,43,44,45,46,47,48,49,50,51,52,53,54,55,56,58,59].

The prevalence with microscopy methods varied between 0.9% (C.I. 0–1.9%) [47] and 100% (C.I. 100–100%) [40]. Overall, of the 23 investigations that used microscopy, 8 papers showed more than 25.0% prevalence of *Giardia* in Colombian individuals [39,40,42,48,49,52,55,59] (Figure 4).

## 4. Discussion

### 4.1. Epidemiology of Giardia in Colombia

A large variety of factors such as geographic–climatic conditions, unequal distribution of resources, unfavorable socioeconomic indicators, and inadequate sanitary indicators influence the transmission of parasitic diseases, particularly Giardiasis, among the Colombian individuals [52,60]. People living in rural areas and children are usually the individuals most exposed to health risks derived from *Giardia* infection [51]. In this sense, Peña-Quistial et al. [56] studied children belonging to disadvantaged migrant populations in the mountain area of the Valle del Cauca and pointed out that the lack of drinking water and a sewage system could be the main detonators of parasitic diseases. Hernández et al. [52] indicated that all the children in their study group, in the Department of Cundinamarca at 100 km from Bogota, were found to be infected by parasites; the microscopic examination revealed that *Giardia* was the most prevalent protozoa (39.1%); the molecular analysis, conducted on a total of 14 *Giardia* positive samples, allowed the authors to identify the presence of subassemblages AI, AII (the most frequent subassemblages), BIII, BIV, BIII/BIV, and a mixed subassemblage AII + BIII. Also, the results of Sánchez et al. [46], who studied the prevalence of intestinal parasites among indigenous children from the Colombian Amazon basin, go in the same direction; the authors attributed the contamination of public water and close contact with domestica and wild animals in the Amazon region with the presence of AI *Giardia* subassemblage.

Ramírez et al. [44], through specific molecular markers, identified in a reliable manner the assemblage B in a great proportion of Cundinamarca children and the assemblage A in a few samples; subsequently, the subassemblages were described as AI, AII, BIII, and BIV.

Fecal samples, collected from children or teenagers living in central and southwest Colombian regions, were analyzed by Avendaño et al. [50], who allocated the *Giardia* assemblages principally to B and to a lesser extent A; the authors suggested a basic transmission among the children attending educational establishments and individuals from urban areas. Also, Muñoz Salas et al. [58] reported that around 13% of schoolchildren, between two and ten years of age, in the department of Atlántico, were infected by *Giardia* protozoa, but the genotypes A and B did not show an association with their nutritional status.

Higuera et al. [55] collected and analyzed 649 stool samples from adults and children in different regions of Colombia and showed the different performances between molecular analysis and microscopic examination; using molecular detection by PCR, 43.1% of samples tested positive for *Giardia* while through microscopy only a quarter of the samples (25.4%) were assessed for *Giardia*. Citizens living in the Caribbean region were more exposed to parasitic infection, particularly in the Bolivar department that registered the highest prevalence of *Giardia* equivalent to 89.5% of the analyzed samples. Finally, the main assemblages identified were BIV and BIII; in some cases, assemblages AII, D, and G were encountered.

Villalba-Vizcaíno et al. [49], looking to obtain frequency and circulating genotypes of *Giardia* in the Colombia Caribbean coast, analyzed fecal samples of citizens from Cartagena de Indias and Santa Marta; all the samples from Santa Marta were molecularly characterized as assemblage A of *Giardia*, while in Cartagena the presence of assemblages A and B has been confirmed.

An interesting study case of Espinosa Aranzales et al. [47] reported a low prevalence of pathogenic intestinal parasites such as *Giardia* in pregnant women in the districts of Bogota; nevertheless, the authors highlighted the need for educational campaigns aimed at the poorest and most marginalized groups in the capital city to disrupt transmission routes for prevalent parasites.

More authors, including Boeke et al. [38], Villafañe-Ferrer and Pinilla-Pérez. [45], Giraldo-Ospina et al. [48], Villamizar et al. [54], Kann et al. [57], and Vásquez et al. [59], highlighted that the *Giardia* infection, genotype principally as assemblage A and B, represents a serious problem of public health in Colombia.

Giardiasis, defined by the World Health Organization (WHO) as a neglected tropical disease, represents a serious concern in public health not only for Colombians but for all people living in Latin America [61]. Annually, approximately 200 million people are estimated to develop Giardiasis symptoms in the developing countries [62]. Studies conducted in Mexico have shown that humans, living in rural areas, are mostly infected by *Giardia* assemblage A [63,64]; while, as reported by Lebbad et al. [65] and Minvielle et al. [66], in Nicaragua and Argentina the assemblage B was dominant.

Similar to Colombia, the *Giardia* assemblages A, B, and A + B were dominant in Cuba; these specific assemblages were detected in groups of children living in La Habana and rural inhabitants in the central region of the country [67,68,69,70]. Brazilian and Colombian indigenous groups, with poor sanitation and unsafe water, are particularly exposed to health risks [71] and specifically to *Giardia* infection [72]; according to Coelho et al. [73], the rate of Giardiasis in the adults and children belonging to Amazonian communities ranging from 44.8 to 52.9%. Köster et al. [74], who investigated the *Giardia* prevalence among the indigenous of the Brazilian Amazonian region, have typified the assemblages as A, B, and A + B, while the dominant subassemblages were AII, AIII, BIII, and BIV; these results coincide with those reported by Sanchez et al. [46], who studied rural people in the Colombia Amazonia. According to Merchán Garzón et al. [19], the prevalence *Giardia* spp. reached up to 63% of individuals in the indigenous and black communities of Colombia.

Many authors [75,76,77,78,79,80,81], indicated that PCR-based methods for the laboratory diagnosis of Giardiasis showed excellent specificity and sensitivity, compared with antigen detection, and microscopy.

### 4.2. Microscopic vs Molecular Methods

Most reports on intestinal protozoan pathogens such as *Giardia* among Colombian population have focused exclusively on microscopic detection [51]. However, many authors have shown substantial differences in detection rates using molecular methods, which also allow to identify cryptic species and their genotypes [46,47,52,54,55,58]. There are clear benefits and additional value in using complementary molecular techniques for molecular epidemiological studies [46,55]; specific marker may help to improve knowledge of the transmission dynamics of intestinal parasites and to establish better prevention campaigns [81].

Classical microscopy is used routinely for *Giardia* cyst detection in water, food, stool, and tissue samples [82,83]. Classical microscopy technique is considered the gold standard for the diagnosis of Giardiasis. However, this technique is subject to subjective interpretation by the observer [84], in addition, the sensitivity is low when only one sample is analyzed, particularly if there is a low density of parasites or if the excretion of *Giardia* cysts is intermittent [85]. Despite this, the sensitivity can be increased if other diagnostic techniques are added [86,87]. Nevertheless, the microscopy remains the mainstay of *Giardia* diagnosis despite its limitations, specially in the developing countries located in Latin America, Asia, and Sub-Saharan Africa with low or middle economic resources and poor access to health facilities [33,76,88].

For the routine medical diagnosis of Giardiasis, it is recommended to combine traditional microscopy with a stool concentration method, thus increasing sensitivity. Immunological and molecular methods are recommended as complementary tests to the traditional microscopy technique [33].

The detection of pathogenic enteroparasites based on DNA analysis offers greater sensitivity and robustness, in addition to enabling the identification and/or characterization of genetic variants. The information obtained is very useful for epidemiological surveillance in the event of an outbreak [89].

PCR-based techniques are powerful molecular tools that make it possible to obtain numerous copies of a desired DNA fragment for the detection of a molecular target or for its subsequent characterization. PCR-based assays have been widely adopted for the detection of *Giardia* in various environments [90,91,92]. These techniques are adaptable and allow the automated processing of large numbers of samples in a short time [93]. Phylogenetic analysis of the *18S rDNA* gene of *Giardia* might reveal significant intraspecies diversity, and, at the same time, highlight the danger of zoonoses from specific assemblages [94]. These techniques require their own standardization, expensive instrumentation, and consumables.

The PCR classifies the genetic variants of *Giardia* [33,95]; the genes most used for this purpose are the small subunit (*SSU*) encoding ribosomal RNA, glutamate dehydrogenase (*gdh*), triosaphosphate isomerase genes (*tpi*), and ß-giardin (*bg*—a protein in the adhesive disc of *Giardia*) [33].

Nested PCR is widely used in the detection of *Giardia* [96,97,98,99]. It is characterized using two sets of primers. The first set binds to sequences slightly outside the target DNA, then this amplified fraction serves as the basis for the second set of primers, this variant of the technique allows increasing sensitivity [91,100].

Polymorphic length restriction fragment-based PCR (PCR-RFLP) uses specific primer sets for the selective amplification of different regions of the genome. The amplification product is then subjected to enzymatic digestion to characterize and classify *Giardia* genetic variants based on the number and size of fragments produced [101,102]. This technique is also frequently used in *Giardia* characterization studies.

Quantitative PCR (qPCR) has had a wide field of application in the detection and quantification of pathogens in environmental and clinical samples [103,104,105]. This variant of conventional PCR offers the advantage of being able to follow the amplification process in real time and thus being able to calculate the number of copies along the process.

The qPCR is widely used in the detection of pathogens due to its great sensitivity and savings in time and effort, in addition there are different variants for this technique such as Molecular Beacon probes, Taqman probes, Scorpion probes, FRET probes, and intercalating dyes such as SYBR Green, that alone or in combination with other techniques have been used for the detection, quantification, and characterization of *Giardia* [92,106,107,108,109].

The choice of the most appropriate technique depends on the objective of the investigation and the type of sample analyzed. The detailed understanding of the foundation of the molecular tool provides the opportunity to make pertinent adjustments when necessary [89].

### 4.3. Burden and Perspective to Colombia

Colombia has faced numerous barriers in improving healthcare for its citizens due to both its topography with wide-ranging landscapes and socioeconomic inequity [110]. The internal armed conflict, lasted over fifty years, produced one of the largest internally displaced populations in the world [111]; it has been an amplifier of social inequalities limiting public health access especially for the weakest groups in society such as the indigenous, farmers, and children.

The Colombian health system has been able to react to these difficulties and taken giant steps to ensure better access to public health and adequate medical care for all citizens [112]; nevertheless, the high burden of numerous neglected tropical diseases such as Giardiasis negatively affect the lives of people with low incomes.

Most of the selected studies of this systematic review were developed in the central region and in the north of the country [38,39,40,42,43,45,49,53,55,57,58,59]; only a few studies have been conducted on southern populations [46,48,54,56]. Census or high-quality research could make up for this data gap by providing reliable values for taking decisions and any eventually encountered, specific *Giardia* assemblages and subassemblages.

In addition, efforts to ensure safe drinking water, sufficient sanitation, and sewage systems in the poorest departments of the country such as Amazonas, Putumayo, Vaupés, and Caquetá are promptly required. Low levels of schooling and a significant food risk can increase the consequences of gastrointestinal infections in the most vulnerable groups in society. Valid measures of food security could form a key component to protect citizens from gastrointestinal diseases, especially *Giardia* infection.

The Colombian healthcare system needs a multidisciplinary management to eradicate *Giardia* spp. infection. Microbiology analysis such classical microscopy and molecular techniques should be integrated with projects in the fields of human and social sciences [34]. Educational and awareness campaigns could be key elements to educate Colombian individuals in correct sanitary hygiene and prevent the *Giardia* spp. infection and distribution of other tropical diseases.

## 5. Conclusions

The prevalence of human Giardiasis between 2006 and 2022 in 22 departments of central and western Colombia was 0.9–48.1% when using classical microscopy and 4.2–87.0% using PCR. Study areas included urban and rural, but due to differences found between the different publications, it was not possible to generalize the type of area associated with the highest prevalence. Apparently, the difference becomes less noticeable when conditions of poverty and deficiencies in public services prevail in urban settlements.

In Colombia, assemblages A and B are present in humans. Two outliers of assemblages D and G are also reported, but further studies are needed to confirm the information. The predominant subassemblages were AII, BIII, and BIV.

## Figures and Tables

**Table 1:** Main characteristic of included studies.

Reference	Quartile SJR	Location	Rural/Urban	Collection Period	Target Population	Age (years)	Quality
Arias et al., 2010 [37]	Q4	Quindío	Rural	2008	Children	2–5	Moderate
Boeke et al., 2010 [38]	Q2	Bogotá	Urban	2006	School Children	5–12	High
Londoño Alvarez et al., 2010 [39]	Q4	Atlántico	Urban	2004	Children	2–6	Moderate
Arroyo-Salgado et al., 2014 [40]	Q4	Bolívar Sucre	Urban	2009	Children	<7	Moderate
Rodriguez et al., 2014 [41]	Q3	Tolima	Urban	2009	Children	1–5	Moderate
Espinosa-Muñoz et al., 2015 [42]	Q4	Magdalena	Rural	2014	Indigenous	1–93	Moderate
Fillot et al., 2015 [43]	Q4	Atlántico	Urban	2014	Children	1–10	Moderate
Ramírez et al., 2015 [44]	Q2	Cundinamarca	Rural	NR	Children	Under 16	High
Villafañe-Ferrer and Pinilla-Pérez, 2016 [45]	Q4	Bolívar	Rural	NR	Children	2–12	Moderate
Sánchez et al., 2017 [46]	Q1	Amazonas	Rural	NR	Children	Under 15	High
Espinosa Aranzales et al., 2018 [47]	Q1	Bogotá	Urban	2015–2016	Pregnant Women	14–43	High
Giraldo-Ospina et al., 2018 [48]	Q3	Valle del Cauca	Rural	2015–2017	Children	1–10	Moderate
Villalba-Vizcaíno et al., 2018 [49]	Q3	Bolívar Magdalena	Urban	NR	Children Adults	0–80	Moderate
Avendaño et al., 2019 [50]	Q2	Bogotá Valle del Cauca Nariño	Rural Urban	2014	Children Teenagers	1–19	High
Carvajal-Restrepo et al., 2019 [51]	Q3	Antioquia Cauca Chocó	Rural	2009–2010	Adults	>18	Moderate
Hernández et al., 2019 [52]	Q1	Cundinamarca	Rural	2017	School Children	1–15	High
Pedraza et al., 2019 [53]	Q4	Bolívar	Urban	NR	Children	2–5	Moderate
Villamizar et al., 2019 [54]	Q1	Cauca	Rural Urban	NR	School Children	1–5	High
Higuera et al., 2020 [55]	Q1	Amazonas Antioquia Bolívar Boyacá Caldas Casanare Cauca Córdoba Cundinamarca Guainía Quindío Risaralda Tolima	Rural Urban	NR	Children Adults	1–70	High
Peña-Quistial et al., 2020 [56]	Q1	Valle del Cauca	Rural	2019	Children	1–12	High
Kann et al., 2022 [57]	Q2	Cesar Guajira	Rural	2014–2018	Indigenous	1–20	High
Muñoz Salas et al., 2022 [58]	Q3	Atlántico	Rural	2017	School Children	2–10	Moderate
Vásquez et al., 2022 [59]	Q4	Córdoba	Rural	2017–2018	Children	1–10	Moderate

NR: not reported.

**Table 2:** Characteristics of molecular test used in the selected studies.

Reference	Samples Analyzed	Replica Number (Mx)	Concentration Method	DNA Extraction Method	Detection Method	Genes Investigated	Assemblages/Subassemblages
Arias et al., 2010 [37]	SS	3	Ritchie concentration technique	NR	Microscopy	NR	NR
Boeke et al., 2010 [38]	SS	1	Formol-ether technique	NR	Microscopy	NR	NR
Londoño Alvarez et al., 2010 [39]	SS	NR	Ritchie concentration technique	NR	Microscopy	NR	NR
Arroyo-Salgado et al., 2014 [40]	SS	NR	NR	Organic solvents	Microscopy Semi-nested PCR	*tpi*	A, B
Rodriguez et al., 2014 [41]	SS	NR	Faust float	QIAmp DNA Stool Mini Kit (Qiagen, Hilden, Germany)	Microscopy PCR-RFLP	*gdh* *bg*	AIII, BIII, BIV
Espinosa-Muñoz et al., 2015 [42]	SS	1	Mini Parasep SF fecal parasite concentrator	NR	Microscopy	NR	NR
Fillot et al., 2015 [43]	SS	NR	Ritchie concentration technique	NR	Microscopy	NR	NR
Ramírez et al., 2015 [44]	SS	NR	Ritchie concentration technique Kato-katz Richie-Frick	QIAmp DNA Stool Mini Kit (Qiagen, Hilden, Germany)	Microscopy Nested and semi-nested PCR	*tpi* *gdh* *SSU rDNA*	A, B, A + B, AI, AII, BIII, BIV
Villafañe-Ferrer and Pinilla-Pérez, 2016 [45]	SS	3	Formol-ether technique	NR	Microscopy	NR	NR
Sánchez et al., 2017 [46]	SS	NR	NR	Norgen Stool DNA isolation kit (Norgen Biotek Corporation, Thorold, Canada)	Microscopy qPCR	*tpi* *gdh*	AI, AII, BIII, BIV
Espinosa Aranzales et al., 2018 [47]	SS	1–2	Formol-ether technique	Norgen Stool DNA isolation kit (Norgen Biotek Corporation, Thorold, Canada)	Microscopy qPCR	*16S rRNA*	NR
Giraldo-Ospina et al., 2018 [48]	SS	NR	Ritchie concentration technique Formol-ether technique	NR	Microscopy	NR	NR
Villalba-Vizcaíno et al., 2018 [49]	SS	NR	Diethyl-ether method	ISOLATE II Genomic DNA Kit Cat.: 137 BIO-52066 (Bioline)	MicroscopyPCR	*tpi* *gdh* *bg*	A
Avendaño et al., 2019 [50]	SS	1	Biphasic sedimentation	QIAmp DNA Stool Mini Kit (Qiagen, Hilden, Germany)	MicroscopyNested PCR	*tpi* *bg* *SSU* *rRNA*	A, AII, B
Carvajal-Restrepo et al., 2019 [51]	SS	NR	Formalin-ethyl acetate technique	NR	Microscopy	NR	NR
Hernández et al., 2019 [52]	SS	1	Mini Parasep SF fecal parasite concentrator	QIAamp^®^ Fast DNA Stool Mini Kit (Qiagen, Hilden, Germany)	Microscopy, Nested and Semi-nested PCR	*tpi* *gdh* *bg*	AI, AII, BIII, BIV, AII + BIII
Pedraza et al., 2019 [53]	SS	NR	NR	NR	Microscopy	NR	NR
Villamizar et al., 2019 [54]	SS	NR	Ritchie concentration technique Kato-katz technique	Norgen Stool DNA isolation kit (Norgen Biotek Corporation, Thorold, Canada)	Microscopy qPCR	*tpi* *gdh*	AII, BIII, BIV, D
Higuera et al., 2020 [55]	SS	NR	Ritchie concentration technique	Norgen Stool DNA isolation kit (Norgen Biotek Corporation, Thorold, Canada)	Microscopy PCR	*tpi* *gdh*	AII, BIII, BIV, D, G
Peña-Quistial et al., 2020 [56]	SS	NR	Sheather technique Kato-katz	NR	Microscopy	NR	NR
Kann et al., 2022 [57]	SS	NR	NR	QIAmp DNA Stool Mini Kit (Qiagen, Hilden, Germany)	PCR	*SSU* *rRNA*	NR
Muñoz Salas et al., 2022 [58]	SS	3	Ritchie concentration technique	AccuPrept Stool Genomic DNA kit (BioNeer Corp., Munpyeong-seo, Republic of Korea)	Microscopy PCR	*bg*	A, B, A + B
Vásquez et al., 2022 [59]	SS	NR	NR	NR	Microscopy	NR	NR

SS: stools samples; NR: not reported; *bg*: beta-giardin; *gdh*: glutamate dehydrogenase; *tpi*: triose phosphate isomerase; *SSU* *rDNA*: small subunit *rDNA*; PCR: polymerase chain reaction; qPCR: quantitative polymerase chain reaction.

## Data Availability

Not applicable.

## Supplementary Materials

The following supporting information can be downloaded at: https://www.mdpi.com/article/10.3390/tropicalmed7100325/s1, Table S1: PRISMA Checklist, Table S2: Quality assessment of included studies.

## References

[1] Morrison H.G., McArthur A.G., Gillin F.D., Aley S.B., Adam R.D., Olsen G.J., Best A.A., Cande W.Z., Chen F., Cipriano M.J. (2007). Genomic minimalism in the early diverging intestinal parasite Giardia lamblia. Science.

[2] Einarsson E., Ma’ayeh S., Svärd S.G. (2016). An up-date on Giardia and giardiasis. Curr. Opin. Microbiol..

[3] Muadica A.S., Balasegaram S., Beebeejaun K., Köster P.C., Bailo B., Hernández-de-Mingo M., Dashti A., Dacal E., Saugar J.M., Fuentes I. (2021). Risk associations for intestinal parasites in symptomatic and asymptomatic schoolchildren in central Mozambique. Clin. Microbiol. Infect..

[4] Thompson R.C.A., Hopkins R.M., Homan W.L. (2000). Nomenclature and genetic groupings of *Giardia* infecting mammals. Parasitol. Today.

[5] Thompson R.C.A., Ash A. (2016). Molecular epidemiology of Giardia and Cryptosporidium infections. Infect. Genet. Evol..

[6] Thompson R.C.A., Ash A. (2019). Molecular epidemiology of Giardia and Cryptosporidium infections—What’s new?. Infect. Genet. Evol..

[7] Adam R.D. (2021). Giardia duodenalis: Biology and Pathogenesis. Clin. Microbiol. Rev..

[8] Muadica A.S., Köster P.C., Dashti A., Bailo B., Hernández-de-Mingo M., Reh L., Balasegaram S., Verlander N.Q., Ruiz Chércoles E., Carmena D. (2020). Molecular diversity of Giardia duodenalis, *Cryptosporidium* spp. and *Blastocystis* sp. in asymptomatic school children in Leganés, Madrid (Spain). Microorganisms.

[9] Dacal E., Saugar J.M., de Lucio A., Hernández-de-Mingo M., Robinson E., Köster P.C., Aznar-Ruiz-de-Alegría M.L., Espasa M., Ninda A., Gandasegui J. (2018). Prevalence and molecular characterization of *Strongyloides stercoralis*, *Giardia duodenalis*, *Cryptosporidium* spp. and *Blastocystis* spp. isolates in school children in Cubal, Western Angola. Parasit. Vectors.

[10] Daniels M.E., Shrivastava A., Smith W.A., Sahu P., Odagiri M., Misra P.R., Panigrahi P., Suar M., Clasen T., Jenkins M.W. (2015). Cryptosporidium and Giardia in humans, domestic animals, and village water sources in rural India. Am. J. Trop. Med. Hyg..

[11] Li J., Wang H., Wang R., Zhang L. (2017). Giardia duodenalis infections in humans and other animals in China. Front. Microbiol..

[12] Zahedi A., Field D., Ryan U. (2017). Molecular typing of Giardia duodenalis in humans in Queensland—First report of assemblage E. Parasitology.

[13] Pipiková J., Papajová I., Majláthová V., Šoltys J., Bystrianska J., Schusterová I., Vargová V. (2020). First report on Giardia duodenalis assemblage F in Slovakian children living in poor environmental conditions. J. Microbiol. Immunol. Infect..

[14] Certad G., Viscogliosi E., Chabé M., Cacciò S.M. (2017). Pathogenic mechanisms of Cryptosporidium and Giardia. Trends Parasitol..

[15] Buret A.G., Cacciò S.M., Favennec L., Svärd S. (2020). Update on Giardia: Highlights from the seventh International Giardia and Cryptosporidium Conference. Parasite.

[16] Feng Y., Xiao L. (2011). Zoonotic potential and molecular epidemiology of Giardia species and giardiasis. Clin. Microbiol. Rev..

[17] Savioli L., Smith H., Thompson A. (2006). Giardia and Cryptosporidium join the ‘Neglected Diseases Initiative’. Trends Parasitol..

[18] Sotelo-Muñoz N.F., Vásquez-Arteaga L.R., González-Fernández D., Marín-Agudelo N.D., González-Cuellar F.E., Montero-Carvajal J.B., Palechor-García M.E. (2017). Situation of intestinal parasitism in preschools of a state child’s home in Popayan, Colombia. Med. Lab..

[19] Merchán Garzón M.C., Ordóñez Vásquez A., Bernal Villegas J., Suárez Obando F. (2016). Estimación de la frecuencia de infección por Giardia intestinalis en comunidades indígenas y afros de Colombia: Estudio de corte trasversal. Medicina.

[20] Gaviria A., Davila C., Ruiz F., Burgos G. (2016). Informe al Congreso de la República 2015-2016: Sector administrativo de salud y protección social. Ministerio de Salud y Protección Social.

[21] Arrivillaga M. (2021). Assesing Health Services in Colombia: Development of a Conceptual Framework and Measurement tools based on primary data. SAGE Open.

[22] Restrepo J., Zambrano A., Vélez M., Ramírez Gómez M. Health insurance as a strategy for access: Streamlined facts of the Colombian Health Care Reform. Universidad del Rosario **2007**, 1–25. https://repository.urosario.edu.co/bitstream/handle/10336/11016/2783.pdf.

[23] Tovar-Cuevas L.M., Arrivillaga-Quintero M. (2014). State of the art in access to health services research in Colombia, 2000–2013: A systematic review. Rev. Gerencia y Politicas de Salud.

[24] Rodríguez-Morales A.J., Granados-Álvarez S., Escudero-Quintero H., Vera-Polania F., Mondragon-Cardona A., Díaz-Quijano F.A., Sosa-Valencia L., Lozada-Riascos C.O., Escobedo A.A., Liseth O. (2016). Estimating and mapping the incidence of giardiasis in Colombia, 2009–2013. Int. J. Infect. Dis..

[25] Bedoya-Arias H.A., Cortés-Puentes P.A., Ramírez-Echeverri M., Montenegro-Jurado H.S., Hernández-Vanegas N., Zapata-Orozco J.M., Cardona-Ospina J.A., Escobedo A.A., Rodriguez-Morales A.J. (2018). Estimating the burden of disease and the economic costs attributable to giardiasis in Colombia, 2009–2016. Int. J. Infect. Dis..

[26] Sarker A.R., Sultana M., Mahumud R.A., Ali N., Huda T.M., Salim Uzzaman M., Haider S., Rahman H., Islam Z., Khan J. (2018). Economic costs of hospitalized diarrheal disease in Bangladesh: A societal perspective. Glob. Health Res. Policy..

[27] Barnes C., Ashton J.J., Borca F., Cullen M., Walker D.M., Beattie R.M. (2020). Children and young people with inflammatory bowel disease attend less school than their healthy Peers. Arch. Dis. Child..

[28] Topal F., Camyar H., Saritas Yuksel E., Gunay S., Topal F., Gür E.Ö. (2020). Work productivity loss in inflammatory bowel disease patients in Turkey. Gastroenterol. Res. Pract..

[29] Campbell S.J., Nery S.V., D’Este C.A., Gray D.J., McCarthy J.S., Traub R.J., Andrews R.M., Llewellyn S., Vallely A.J., Williams G.M. (2016). Water, sanitation and hygiene related risk factors for soil-transmitted helminth and Giardia duodenalis infections in rural communities in Timor-Leste. Int. J. Parasitol..

[30] Yazdani H., Sharafi S.M., Yousefi H., Hadipur M., Sepahvand A., Darani H.Y. (2016). Diagnosis of Giardia duodenalis infection using dot blot in comparison with microscopy. Infect. Disord. Drug Targets..

[31] Chourabi M., Boughattas S., Abdallah A.M., Ismail A., Behnke J.M., Al-Mekhlafi H.M., Abu-Madi M. (2021). Genetic Diversity and Prevalence of *Giardia duodenalis* in Qatar. Front. Cell. Infect. Microbiol..

[32] Sarzosa M., Graham J.P., Salinas L., Trueba G. (2018). Potential zoonotic transmission of Giardia duodenalis in semi-rural communities near Quito, Ecuador. Int. J. Appl. Res. Vet. Med..

[33] Hooshyar H., Rostamkhani P., Arbabi M., Delavari M. (2019). Giardia lamblia infection: Review of current diagnostic strategies. Gastroenterol. Hepatol. Bed. Bench..

[34] Duque S., Arévalo A., Nicholls R.S. (2021). La Parasitología en Colombia: Una visión panorámica. Biomedica.

[35] Moher D., Liberati A., Tetzlaff J., Altman D.G., PRISMA Group (2009). Preferred reporting items for systematic reviews and meta-analyses: The PRISMA statement. PLoS Med..

[36] Munn Z., Moola S., Lisy K., Riitano D., Tufanaru C. (2015). Methodological guidance for systematic reviews of observational epidemiological studies reporting prevalence and cumulative incidence data. Int. J. Evid. Based Healthc..

[37] Arias J.A., Guzmán G.E., Lora-Suárez F.M., Torres E., Gómez J.E. (2010). Prevalencia de protozoos intestinales en 79 niños de 2 a 5 años de edad de un hogar infantil estatal en Circasia, Quindío. Infectio.

[38] Boeke C.E., Mora-Plazas M., Forero Y., Villamor E. (2010). Intestinal protozoan infections in relation to nutritional status and gastrointestinal morbidity in Colombian school children. J. Trop. Pediatr..

[39] Londoño Alvarez J.C., Hernández A.P., Vergara Sánchez C. (2010). Parasitismo intestinal en hogares comunitarios de dos municipios del departamento del Atlántico, norte de Colombia. Bol. Malariol. Salud Ambient..

[40] Arroyo-Salgado B., Buelvas-Montes Y., Villalba-Vizcaíno V., Salomón-Arzuza O. (2014). Caracterización genética por reacción en cadena de la polimerasa de Giardia intestinalis en muestras de humanos y perros del Caribe colombiano. Enferm. Infecc. Microbiol. Clin..

[41] Rodríguez V., Espinosa O., Carranza J.C., Duque S., Arévalo A., Clavijo J.A., Urrea D.A., Vallejo G.A. (2014). Genotipos de Giardia duodenalis en muestras de niños de las guarderías del Instituto Colombiano de Bienestar Familiar y de perros en Ibagué, Colombia. Biomedica.

[42] Espinosa-Muñoz D.Y., Gómez-Gómez N.E., Polanco L.C., Cardona- Osorio J.A.C., Ríos-Arias L. (2015). Prevalencia de parasitismo intestinal en la comunidad Seminke del resguardo indígena Wiwa de la Sierra Nevada de Santa Marta, 2014. Arch. de medicina.

[43] Fillot M., Guzman J., Cantillo L., Gómez L., Sánchez Majana L., Acosta B.M., Sarmiento-Rubiano L.A. (2015). Prevalencia de parásitos intestinales en niños del Área Metropolitana de Barranquilla, Colombia. Rev. Cubana Med. Trop..

[44] Ramírez J.D., Heredia R.D., Hernández C., León C.M., Moncada L.I., Reyes P., Pinilla A.E., Lopez M.C. (2015). Molecular diagnosis and genotype analysis of Giardia duodenalis in asymptomatic children from a rural area in central Colombia. Infect. Genet. Evol..

[45] Villafañe-Ferrer L.M., Pinilla-Pérez M. (2016). Intestinal parasites in children and soil from Turbaco, Colombia and associated risk factors. Rev, Salud Publica (Bogota).

[46] Sánchez A., Munoz M., Gómez N., Tabares J., Segura L., Salazar Á., Restrepo C., Ruíz M., Reyes P., Qian Y. (2017). Molecular Epidemiology of Giardia, Blastocystis and Cryptosporidium among Indigenous Children from the Colombian Amazon Basin. Front. Microbiol..

[47] Espinosa Aranzales A.F., Radon K., Froeschl G., Pinzon Rondon A.M., Delius M. (2018). Prevalence and risk factors for intestinal parasitic infections in pregnant women residing in three districts of Bogotá, Colombia. BMC Public Health.

[48] Giraldo-Ospina B., Fontal-Vargas P.A., López-Muñoz D.F., Beltrán-Angarita L., Morales-Jiménez V., Gómez M.N. (2018). Prevalence of intestinal parasites in children of an invasion community in a municipality of Colombia. Int. J. Biol. Biomed. Eng..

[49] Villalba-Vizcaíno V., Buelvas Y., Arroyo-Salgado B., Castro L.R. (2018). Molecular identification of Giardia intestinalis in two cities of the Colombian Caribbean Coast. Exp. Parasitol..

[50] Avendaño C., Ramo A., Vergara-Castiblanco C., Bayona M., Velasco-Benitez C.A., Sánchez-Acedo C., Quílez J. (2019). Occurrence and molecular characterization of Giardia duodenalis in child population from Colombia. Infect. Genet. Evol..

[51] Carvajal-Restrepo H., Orrego-Morales C., Vega-Orrego T., Arango-Arango S., Buitrago-Agudelo D., Maya-Betancourt M.C., Maya-Betancourt V., Restrepo-Álvarez L., Silva-Cáceres N., Suarez-Urquijo S. (2019). Screening for intestinal parasites in adults from three different regions of Colombia. Infectio.

[52] Hernández P.C., Morales L., Chaparro-Olaya J., Sarmiento D., Jaramillo J.F., Ordoñez G.A., Cortés F., Sánchez L.K. (2019). Intestinal parasitic infections and associated factors in children of three rural schools in Colombia. A cross-sectional study. PLoS ONE.

[53] Pedraza B., Suarez H., De-la-Hoz I., Fragoso P. (2019). Prevalencia de parásitos intestinales en niños de 2-5 años en hogares comunitarios de Cartagena de Indias, Colombia. Rev. Chil. Nutr..

[54] Villamizar X., Higuera A., Herrera G., Vasquez-A L.R., Buitron L., Muñoz L.M., Gonzalez-C F.E., Lopez M.C., Giraldo J.C., Ramírez J.D. (2019). Molecular and descriptive epidemiology of intestinal protozoan parasites of children and their pets in Cauca, Colombia: A cross-sectional study. BMC Infect. Dis..

[55] Higuera A., Villamizar X., Herrera G., Giraldo J.C., Vasquez-A L.R., Urbano P., Villalobos O., Tovar C., Ramírez J.D. (2020). Molecular detection and genotyping of intestinal protozoa from different biogeographical regions of Colombia. PeerJ.

[56] Peña-Quistial M.G., Benavides-Montaño J.A., Duque N.J.R., Benavides-Montaño G.A. (2020). Prevalence and associated risk factors of Intestinal parasites in rural high-mountain communities of the Valle del Cauca-Colombia. PLoS Negl. Trop. Dis..

[57] Kann S., Hartmann M., Alker J., Hansen J., Dib J.C., Aristizabal A., Concha G., Schotte U., Kreienbrock L., Frickmann H. (2022). Seasonal patterns of enteric pathogens in Colombian Indigenous People–A more pronounced effect on bacteria than on parasites. Pathogens.

[58] Muñoz Salas K., Barrios A.P., Gonzalez C.M., Macias J.R., Zapata C.V. (2022). Giardia duodenalis genotyping not linked to clinical symptomatology and nutritional status of school-aged children of Soledad and Galapa municipality schools, Atlántico, Colombia. J. Parasitol..

[59] Vásquez D., Drews-Elger K., Saldarriaga-Muñoz P.J., Correa-Sierra S., Gaviria-Gallego D.A., Atehortúa-Salazar S., Valencia N., Cardona-Castro M.C. (2022). Intestinal parasitosis in children from a rural Caribbean area in Colombia. Infectio.

[60] Giraldo-Gómez J.M., Lora F., Henao L.H., Mejía S., Gómez-Marín J.E. (2005). Prevalencia de giardiasis y parásitos intestinales en preescolares de hogares atendidos en un programa estatal en Armenia, Colombia [Prevalence of giardiasis and intestinal parasites in pre-school children from homes being attended as part of a state programme in Armenia, Colombia]. Rev. Salud Publica (Bogota).

[61] Fornace K.M., Senyonjo L., Martin D.L., Gwyn S., Schmidt E., Agyemang D., Marfo B., Addy J., Mensah E., Solomon A.W. (2022). Characterising spatial patterns of neglected tropical disease transmission using integrated sero-surveillance in Northern Ghana. PLoS Negl. Trop. Dis..

[62] Samie A., Tanih N.F., Seisa I., Seheri M., Mphahlele J., ElBakri A., Mbati P. (2020). Prevalence and genetic characterization of Giardia lamblia in relation to diarrhea in Limpopo and Gauteng provinces, South Africa. Parasite Epidemiol. Control..

[63] Eligio-García L., Cortes-Campos A., Cota-Guajardo S., Gaxiola S., Jiménez-Cardoso E. (2008). Frequency of Giardia intestinalis assemblages isolated from dogs and humans in a community from Culiacan, Sinaloa, Mexico using beta-giardin restriction gene. Vet. Parasitol..

[64] García-Cervantes P.C., Báez-Flores M.E., Delgado-Vargas F., Ponce-Macotela M., Nawa Y., De-la-Cruz-Otero M.D., Martínez-Gordillo M.N., Díaz-Camacho S.P. (2017). Giardia duodenalis genotypes among schoolchildren and their families and pets in urban and rural areas of Sinaloa, Mexico. J. Infect. Dev. Ctries..

[65] Lebbad M., Ankarklev J., Tellez A., Leiva B., Andersson J.O., Svärd S. (2008). Dominance of Giardia assemblage B in León, Nicaragua. Acta Trop..

[66] Minvielle M.C., Molina N.B., Polverino D., Basualdo J.A. (2008). First genotyping of Giardia lamblia from human and animal feces in Argentina, South America. Mem. Inst. Oswaldo Cruz.

[67] Jerez Puebla L.E., Núñez F.A., Santos L.P., Rivero L.R., Silva I.M., Valdés L.A., Millán I.A., Müller N. (2017). Molecular analysis of Giardia duodenalis isolates from symptomatic and asymptomatic children from La Habana, Cuba. Parasite Epidemiol. Control.

[68] Jerez Puebla L.E., Núñez F.A., García A.B., Rivero L.R., Millán I.A., Prado R.C. (2017). Prevalence of Giardia duodenalis among children from a central region of Cuba: Molecular characterization and associated risk factors. J. Parasit. Dis..

[69] Jerez Puebla L.E., Núñez-Fernández F.A., Fraga Nodarse J., Atencio Millán I., Cruz Rodríguez I., Martínez Silva I., Ayllón Valdés L., Robertson L.J. (2020). Diagnosis of intestinal protozoan infections in patients in Cuba by microscopy and molecular methods: Advantages and disadvantages. J. Microbiol. Methods.

[70] Jerez Puebla L.E., Núñez Fernández F.A., Fraga J., Rivero L.R., Millán I.A., Valdés L.A., Silva I.M., Müller N., Robertson L.J. (2020). Concordance of Giardia duodenalis assemblages determined by different PCR methodologies in three observational studies in Cuba. Exp. Parasitol..

[71] Flores-Ramírez R., Berumen-Rodríguez A.A., Martínez-Castillo M.A., Alcántara-Quintana L.E., Díaz-Barriga F., Díaz de León-Martínez L. (2021). A review of Environmental risks and vulnerability factors of indigenous populations from Latin America and the Caribbean in the face of the COVID-19. Glob. Public Health.

[72] Sarria-Guzmán Y., Chávez-Romero Y., Bernal J.E., González-Jiménez F.E., Serrano-Silva N., Fusaro C. (2022). Molecular identification of *Giardia* spp. in Latin America: An updated systematic review on reports from 2017 to 2021. J. Infect. Dev. Ctries..

[73] Coelho C.H., Durigan M., Leal D.A.G., Schneider A.B., Franco R.M.B., Singer S.M. (2017). Giardiasis as a neglected disease in Brazil: Systematic review of 20 years of publications. PLoS Negl. Trop. Dis..

[74] Köster P.C., Malheiros A.F., Shaw J.J., Balasegaram S., Prendergast A., Lucaccioni H., Moreira L.M., Lemos L.M.S., Dashti A., Bailo B. (2021). Multilocus Genotyping of Giardia duodenalis in Mostly Asymptomatic Indigenous People from the Tapirapé Tribe, Brazilian Amazon. Pathogens.

[75] Elsafi S.H., Al-Maqati T.N., Hussein M.I., Adam A.A., Hassan M.M., Al Zahrani E.M. (2013). Comparison of microscopy, rapid immunoassay, and molecular techniques for the detection of Giardia lamblia and Cryptosporidium parvum. Parasitol. Res..

[76] Beyhan Y.E., Taş Cengiz Z. (2017). Comparison of microscopy, ELISA, and real-time PCR for detection of Giardia intestinalis in human stool specimens. Turk. J. Med. Sci..

[77] Incani R.N., Ferrer E., Hoek D., Ramak R., Roelfsema J., Mughini-Gras L., Kortbeek T., Pinelli E. (2017). Diagnosis of intestinal parasites in a rural community of Venezuela: Advantages and disadvantages of using microscopy or RT-PCR. Acta Trop..

[78] Hijjawi N., Yang R., Hatmal M., Yassin Y., Mharib T., Mukbel R., Mahmoud S.A., Al-Shudifat A.E., Ryan U. (2018). Comparison of ELISA, nested PCR and sequencing and a novel qPCR for detection of Giardia isolates from Jordan. Exp. Parasitol..

[79] Emisiko J., Shaviya N., Shiluli C., Kiboi N., Wamalwa R., Jumba B., Zablon J., Mambo F., Barasa M. (2020). Comparison of Microscopy and PCR for Detection of Giardia Lamblia and Entamoeba Histolytica in Human Stool Specimens in a Resource Limited Setting in Western Kenya. Ethiop. J. Health Sci..

[80] Dashti A., Köster P.C., Carmena D. (2021). Giardia duodenalis: Detection by Quantitative Real-Time PCR and Molecular Diversity. Methods Mol. Biol..

[81] Weinreich F., Hahn A., Eberhardt K.A., Kann S., Feldt T., Sarfo F.S., Di Cristanziano V., Frickmann H., Loderstädt U. (2022). Comparative Evaluation of Real-Time Screening PCR Assays for Giardia duodenalis and of Assays Discriminating the Assemblages A and B. Microorganisms.

[82] Quintero-Betancourt W., Gennaccaro A.L., Scott T.M., Rose J.B. (2003). Assessment of methods for detection of infectious Cryptosporidium oocysts and Giardia cysts in reclaimed effluents. Appl. Environ. Microbiol..

[83] Soares R., Tasca T. (2016). Giardiasis: An update review on sensitivity and specificity of methods for laboratorial diagnosis. J. Microbiol. Methods.

[84] Koontz F., Weinstock J.V. (1996). The approach to stool examination for parasites. Gastroenterol. Clin. North Am..

[85] El-Nahas H.A., Salem D.A., El-Henawy A.A., El-Nimr H.I., Abdel-Ghaffar H.A., El-Meadawy A.M. (2013). Giardia diagnostic methods in human fecal samples: A comparative study. Cytometry B. Clin. Cytom..

[86] Mank T.G., Zaat J.O., Deelder A.M., van Eijk J.T., Polderman A.M. (1997). Sensitivity of microscopy versus enzyme immunoassay in the laboratory diagnosis of giardiasis. Eur. J. Clin. Microbiol. Infect. Dis..

[87] Al F.D., Kuştimur S., Ozekinci T., Balaban N., Ilhan M.N. (2006). The use of enzyme linked immunosorbent assay (ELISA) and direct fluorescent antibody (DFA) methods for diagnosis of Giardia intestinalis. Turkiye Parazitol. Derg..

[88] Kabir M.H.B., Han Y., Lee S.H., Nugraha A.B., Recuenco F., Murakoshi F., Xuan X., Kato K. (2020). Prevalence and molecular characterization of Cryptosporidium species in poultry in Bangladesh. One Health.

[89] Adeyemo F.E., Singh G., Reddy P., Stenström T.A. (2018). Methods for the detection of Cryptosporidium and Giardia: From microscopy to nucleic acid based tools in clinical and environmental regimes. Acta Trop..

[90] Guy R.A., Payment P., Krull U.J., Horgen P.A. (2003). Real-time PCR for quantification of Giardia and Cryptosporidium in environmental water samples and sewage. Appl. Environ. Microbiol..

[91] Castro-Hermida J.A., González-Warleta M., Mezo M. (2015). *Cryptosporidium* spp. and Giardia duodenalis as pathogenic contaminants of water in Galicia, Spain: The need for safe drinking water. Int. J. Hyg. Environ. Health.

[92] Nguyen T.T., Traub R., Pham P.D., Nguyen H.V., Nguyen K.C., Phung C.D., Dalsgaard A. (2016). Prevalence and molecular characterization of *Cryptosporidum* spp. and *Giardia* spp. in environmental samples in Hanam province, Vietnam. Food Waterborne Parasitol..

[93] Vohra P., Sharma M., Chaudhary U. (2012). A comprehensive review of diagnostic techniques for detection of Cryptosporidium parvum in stool samples. J. Pharm..

[94] Abdelaziz A.R., Sorour S.S.G. (2021). Prevalence and Molecular Characterization of Giardia duodenalis Assemblage D of Dogs in Egypt, and Its Zoonotic Implication. Microbes Infect. Chemother..

[95] Smith H.V., Mank T.G., Lujan H.D., Svard S. (2011). Diagnosis of human Giardiasis. Giardia a Model Organism.

[96] Monis P.T., Saint C.P. (2001). Development of a nested-PCR assay for the detection of *Cryptosporidium parvum* in finished water. Water Res..

[97] Osaki S.C., Soccol V.T., Costa A.O., Oliveira-Silva M.B., Pereira J.T., Procópio A.E. (2013). Polymerase chain reaction and nested-PCR approaches for detecting Cryptosporidium in water catchments of water treatment plants in Curitiba, State of Paraná, Brazil. Rev. Soc. Bras. Med. Trop..

[98] Prystajecky N., Huck P.M., Schreier H., Isaac-Renton J.L. (2014). Assessment of *Giardia* and *Cryptosporidium* spp. as a microbial source tracking tool for surface water: Application in a mixed-use watershed. Appl. Environ. Microbiol..

[99] Ulloa-Stanojlović F.M., Aguiar B., Jara L.M., Sato M.I., Guerrero J.A., Hachich E., Matté G.R., Dropa M., Matté M.H., de Araújo R.S. (2016). Occurrence of *Giardia intestinalis* and *Cryptosporidium* sp. in wastewater samples from São Paulo State, Brazil, and Lima, Peru. Environ. Sci. Pollut. Res. Int..

[100] Nikaeen M., Mesdaghinia A.R., Jeddi T.M., Rezaeian M., Makimura K. (2005). A nested-PCR assay for detection of Cryptosporidium parvum oocysts in water samples. Iran. J. Public Health.

[101] Coupe S., Delabre K., Pouillot R., Houdart S., Santillana-Hayat M., Derouin F. (2006). Detection of *Cryptosporidium*, *Giardia* and *Enterocytozoon bieneusi* in surface water, including recreational areas: A one-year prospective study. FEMS Immunol. Med. Microbiol..

[102] Almeida A., Moreira M.J., Soares S., Delgado M., Figueiredo J., Silva E., Castro A., Cosa J.M. (2010). Presence of *Cryptosporidium* spp. and *Giardia duodenalis* in drinking water samples in the North of Portugal. Korean J. Parasitol..

[103] Singh G., Vajpayee P., Ram S., Shanker R. (2010). Environmental reservoirs for enterotoxigenic Escherichia coli in south Asian Gangetic riverine system. Environ. Sci. Technol..

[104] Hanabara Y., Ueda Y. (2016). A rapid and simple real-time PCR assay for detecting foodborne pathogenic bacteria in human feces. Jpn. J. Infect. Dis..

[105] Singh G., Vajpayee P., Rani N., Amoah I.D., Stenström T.A., Shanker R. (2016). Exploring the potential reservoirs of nonspecific TEM beta lactamase (bla(TEM)) gene in the Indo-Gangetic region: A risk assessment approach to predict health hazards. J. Hazard. Mater..

[106] Jothikumar N., Murphy J.L., Hill V.R. (2021). Detection and identification of *Giardia* species using real-time PCR and sequencing. J. Microbiol. Methods.

[107] Klotz C., Radam E., Rausch S., Gosten-Heinrich P., Aebischer T. (2021). Real-Time PCR for molecular detection of zoonotic and non-zoonotic *Giardia* spp. in wild rodents. Microorganisms.

[108] Squire S.A., Yang R., Robertson I., Ayi I., Ryan U. (2017). Molecular characterization of Cryptosporidium and Giardia in farmers and their ruminant livestock from the Coastal Savannah zone of Ghana. Infect. Genet. Evol..

[109] Uiterwijk M., Nijsse R., Kooyman F., Wagenaar J.A., Mughini-Gras L., Koop G., Ploeger H.W. (2018). Comparing four diagnostic tests for Giardia duodenalis in dogs using latent class analysis. Parasit. Vectors.

[110] Legido-Quigley H., Camacho Lopez P.A., Balabanova D., Perel P., Lopez-Jaramillo P., Nieuwlaat R., Schwalm J.D., McCready T., Yusuf S., McKee M. (2015). Patients’ knowledge, attitudes, behaviour and health care experiences on the prevention, detection, management and control of hypertension in Colombia: A qualitative study. PLoS ONE.

[111] Rentería-Ramos R., Hurtado-Heredia R., Urdinola B.P. (2019). Morbi-Mortality of the Victims of Internal Conflict and Poor Population in the Risaralda Province, Colombia. Int. J. Environ. Res. Public Health..

[112] Lamprea E., García J. (2016). Closing the Gap between Formal and Material Health Care Coverage in Colombia. Health Hum. Rights..

